# Construction of a predictive model of abdominal lymph node metastasis in thoracic esophageal squamous cell carcinoma and preliminary analysis of its effect on target for postoperative radiotherapy

**DOI:** 10.3389/fsurg.2022.1039532

**Published:** 2022-11-16

**Authors:** Hong-Mei Gao, Xue-Yuan Zhang, Wen-Bin Shen, Jin-Rui Xu, You-Mei Li, Shu-Guang Li, Shu-Chai Zhu

**Affiliations:** ^1^ Department of Radiation, Shijiazhuang People’s Hospital, Shijiazhuang, China; ^2^ Department of Radiation Oncology, The Fourth Hospital of Hebei Medical University, Shijiazhuang, China

**Keywords:** esophageal tumor/esophageal squamous cell carcinoma, abdominal lymph node metastasis, mediastinal lymph node metastasis, prediction model, prognosis, postoperative radiotherapy

## Abstract

**Purpose:**

To investigate the influencing factors of abdominal lymph node metastasis in thoracic esophageal squamous cell carcinoma (TESCC), and to construct its predictive model, in order to analyze the targets for postoperative radiotherapy.

**Methods and materials:**

From January 2008 to December 2014, the clinicopathological data of 479 patients who underwent radical resection for esophageal cancer in the Fourth Hospital of Hebei Medical University were collected and retrospectively analyzed. The influencing factors of postoperative abdominal lymph node metastasis were analyzed, and a predictive model was constructed based on their independent influencing factors. Receiver operating characteristic (ROC) curve was utilized to analyze the predictive value of this model; in the meantime, the postoperative locoregional recurrence (LRR) of this group was analyzed.

**Results:**

The postoperative pathology of all patients showed that the lymph node metastasis rate (LNR) was 39.7%, of which the abdominal lymph node metastasis rate was 22.0%. Logistic regression analysis revealed that the patient's lesion location, pN stage, vascular invasion, LND and mediastinal lymph node metastasis were independent risk factors for the positive rate of abdominal lymph nodes after surgery (*P* = 0.000, 0.000, 0.033, 0.000, 0.000). The probability of abdominal lymph node metastasis was Y = e^x^/(1 + e^x^), and X = −5.502 + 1.569 × lesion location + 4.269 × pN stage + 1.890 × vascular invasion + 1.950 × LND-4.248 × mediastinal lymph node metastasis. The area under the ROC curve (AUC) of this model in predicting abdominal lymph node metastasis was 0.962 (95% CI, 0.946–0.977). This mathematical model had a high predictive value for the occurrence of abdominal lymph node metastasis (*P* = 0.000), and the sensitivity and specificity of prediction were 94.6% and 88.3% respectively. The overall survival rate was significantly higher (X^2 ^= 29.178, *P* = 0.000), while abdominal lymph node recurrence rate was lower in patients with negative abdominal lymph nodes than in those with negative lymph nodes (1.4%&7.7%, X^2 ^= 12.254, *P* = 0.000).

**Conclusion:**

The lesion location, pN stage, vascular invasion, LND and mediastinal lymph node metastasis are independent influencing factors of abdominal lymph node metastasis in patients with TESCC. The mathematical model constructed by these indicators can accurately predict abdominal lymph node metastasis, which can help clinicians to choose the targets for postoperative radiotherapy.

## Introduction

Radical surgical resection is the mainstay of treatment for patients with operable esophageal cancer (EC), and the postoperative pathology of patients has shown that positive lymph node is a predictor of poor prognosis (1–3), and due to the complexity of the arrangement of the lymphatic drainage system in the esophageal wall, lymph node metastasis is characterized by certain regularity and incomplete predictability (4). Therefore, it is common that patients with thoracic EC have positive abdominal lymph nodes after surgery (5–7). The prediction of presence or absence of abdominal lymph node metastasis before clinical treatment may affect the choice of surgical approach by the surgeons, and also the choice of the targets for radiotherapy by the radiotherapists (8–10). Therefore, the effective prediction of presence or absence of abdominal lymph node metastasis before treatment of patients with EC has aroused great attention in clinical research. In this study, we aimed to predict the probability of metastasis before clinical treatment by analyzing the relationship between related indicators, including the general clinical data and postoperative pathology of EC patients, and the nature of postoperative abdominal lymph nodes, in order to avoid unnecessary surgical damage or reduce the targets for radiotherapy, and guide the choice of treatment scheme by clinicians. Therefore, we conducted a retrospective analysis of 479 patients undergoing surgery for EC in our research center.

## Materials and methods

### General data

The clinical and pathological data of patients who underwent radical surgery for EC in the Department of Thoracic Surgery, the Fourth Hospital of Hebei Medical University from January 2008 to December 2014 were collected. A total of 479 patients (343 males and 136 females) were enrolled and received retrospective analysis. The range of age was from 41 to 75 years with a median age of 60 years. The length of esophageal lesion by preoperative barium contrast examination was 1.0–10.0 cm, with a median of 5.0 cm. According to the 8th edition of AJCC/NCCN for TNM classification for EC after surgery, the number of patients with lesions located in the upper, middle, and lower thoracic segments was 39, 337, and 103, respectively; the number of patients with pathological stages T1, T2, T3, and T4 were 13, 105, 345, and 16, respectively, while the number of patients with pathological stages N0, N1, N2, and N3 was 289, 156, 29, and 5, respectively. The number of lymph nodes dissected intraoperatively ranged from 9 to 32, with a median of 12. Postoperative pathology showed moderate/well differentiated squamous cell carcinoma in 414 patients, and undifferentiated or poorly differentiated squamous cell carcinoma in the remaining 65 patients.

### Inclusion and exclusion criteria

Inclusion criteria were as follows: R0 resection; squamous cell carcinoma by postoperative pathology; no neoadjuvant (chemo)radiotherapy before surgery; no adjuvant radiotherapy after surgery; patients with complete clinicopathological data. Exclusion criteria were as follows: non-R0 resection; cervical or esophagogastric junction EC; non-squamous organelles by postoperative pathology; preoperative neoadjuvant (chemo)radiotherapy; postoperative adjuvant radiotherapy; accompanied by a history of other malignant tumors or metastatic carcinoma of the esophagus; incomplete clinical and/or pathological data.

### Surgical approach

The surgical approach of the patients in the group was carried out through a posterolateral incision in the sixth intercostal space of the left thorax. The stomach was freed through a diaphragm incision, and a tubular stomach was made to be used as a substitute organ for the esophagus. Patients with middle and lower thoracic EC underwent esophagogastric anastomosis, while those with upper thoracic EC underwent esophagogastric anastomosis through a left neck incision.

### Calculation of lymph node metastasis rate (LNR)

LNR = total number of patients with positive lymph node metastasis/total number of cases. Calculation of lymph node metastasis degree (LND): LND = the number of positive lymph nodes per patient/the number of lymph nodes dissected per patient.

### Definition of locoregional recurrence (LRR)

LRR after treatment mainly included recurrence of anastomoses and locoregional lymph nodes. Among them, the recurrence of anastomosis needs to be confirmed pathologically by electronic gastroscopy, and superficial lymph node metastasis was confirmed pathologically by needle biopsy. The diagnosis of lymph node metastasis in the remaining regions was confirmed by CT, MRI, PET/CT or B-ultrasound. Regional lymph node metastasis included lymph node metastasis in the supraclavicular area, mediastinum and abdominal cavity.

### Follow-up

The follow-up methods included telephone follow-up, outpatient review, and medical records. Follow-up was initiated from the date of surgery until December 31, 2019. The patient were reviewed every 3–6 months in the first year, and every 6–12 months thereafter. Of them, 13 cases were lost to follow-up, and the rate of loss to follow-up was 2.7%. The patients lost to follow-up were regarded as deaths on the date of the last follow-up.

### Statistical analysis

The SPSS19.0 statistical software was used for statistical analysis. Chi-square test was used for the univariate analysis of the relationship between abdominal lymph node metastasis and various variables. Logistic regression analysis was performed for the multivariate analysis of factors affecting abdominal lymph node metastasis. Significant variables in multivariate analysis were used to establish a predictive mathematical model. The predicted probability value was used as the test variable, and abdominal lymph node metastasis served as the state variable. The receiver operating characteristic (ROC) curve was drawn to evaluate the predictive ability of the model. COX multivariate analysis model was employed for analysis of the patient's prognosis. The difference was statistically significant when *P* < 0.05.

## Results

### Lymph node metastasis by postoperative pathology

Among the 479 patients, 190 had lymph node metastasis (LNR = 39.7%), including 85 (17.7%) with only mediastinal lymph node metastasis, 63 (13.2%) with only mediastinal lymph node metastasis, and 42 (8.8%) with mediastinal and abdominal lymph node metastasis. The LND of all patients was 0%–100.0%, and LND of 190 patients with positive lymph nodes was 4.3%–100.0%, with a median of 20.0%.

### Analysis results of the influencing factors of abdominal lymph node metastasis by postoperative pathology

The clinical, pathological and other related indicators and abdominal lymph node metastasis of patients were analyzed by univariate analysis. The results showed that the patient's lesion location, pN stage, vascular invasion, mediastinal lymph node metastasis and LND were all significant related factors of abdominal lymph node metastasis (X^2 ^= 25.259, 215.431, 11.447, 12.554, 205.564, *P* = 0.000, 0.000, 0.001, 0.000, 0.000), as shown in Table 1. The presence or absence of abdominal lymph node metastasis was used as the dependent variable, and the indicators with *P* < 0.05 in the univariate analysis were used as the independent variables for Logistic regression analysis. The results of multivariate analysis suggested that the patient's lesion location, pN stage, vascular invasion, LND and mediastinal lymph node metastasis were independent factors affecting abdominal lymph node metastasis (*P* = 0.000, 0.000, 0.033, 0.000, 0.000), as shown in Table 2.

**Table 1:** Univariate analysis results of factors affecting postoperative abdominal lymph node metastasis in 479 patients with thoracic esophageal squamous cell carcinoma.

Indicators	*N*	Abdominal lymph node metastasis *N* (%)		X^2^	*P*	Indicators	*N*	Abdominal lymph node metastasis *N* (%)		X^2^	*P*
		No	Yes					No	Yes		
Sex				0.085	0.771	T1 + 2	118	91 (77.1)	27 (22.9)		
Male	343	269 (78.4)	74 (21.6)			T3 + 4	361	283 (78.4)	78 (21.6)		
Female	136	105 (77.2)	31 (22.8)			pN stage				215.431	0.000
Age (y)				2.493	0.114	N0	289	289 (100.0)	0 (0.0)		
≤60	232	174 (75.0)	58 (25.0)			N1	156	77 (49.4)	79 (50.6)		
>60	247	200 (81.0)	47 (19.0)			N2 + 3	34	8 (23.5)	26 (76.5)		
Preoperative lymph nodes				2.472	0.116	Vascular invasion				11.447	0.001
No	361	288 (79.8)	72 (20.2)			No	468	370 (79.1)	98 (20.9)		
Yes	118	86 (72.9)	32 (27.1)			Yes	11	4 (36.4)	7 (63.6)		
Lesion location				25.259	0.000	No. of lymph nodes dissected				0.002	0.965
Upper thoracic segment	39	39 (100.0)	0 (0.0)			≤12	250	195 (78.0)	55 (22.0)		
Middle thoracic segment	337	270 (80.1)	67 (19.9)			>12	229	179 (78.2)	50 (21.8)		
Lower thoracic segment	103	65 (63.1)	38 (36.9)			Mediastinal lymph nodes				12.554	0.000
Lesion length				0.695	0.404	Negative	301	289 (82.1)	63 (17.9)		
≤5.0	268	213 (29.5)	55 (20.5)			Positive	85	85 (66.9)	42 (33.1)		
>5.0	211	161 (76.3)	50 (23.7)			LND				205.564	0.000
Tumor differentiation				0.059	0.808	0%	289	289 (100.0)	0 (0.0)		
Moderate/Well	414	324 (78.3)	90 (21.7)			>0%, <30%	135	63 (46.7)	72 (53.3)		
No or poor	65	50 (76.9)	15 (23.1)			≥30%	55	22 (40.0)	33 (60.0)		
pT stage				0.084	0.771						

**Table 2:** Multivariate analysis results of factors affecting postoperative abdominal lymph node metastasis in 479 patients with thoracic esophageal squamous cell carcinoma.

Indicators	Grouping and assignment	B	SD	Wald value	*P*	Odds ratio	95.0% CI	
							Lower limit	Upper limit
Lesion location	Upper/Middle/Lower (1/2/3)	1.569	0.450	12.140	0.000	4.801	1.986	11.603
pN stage	N0/N1/N2 + 3 (0/1/2)	4.269	0.508	70.703	0.000	71.420	26.406	193.167
Vascular invasion	No/Yes (1/2)	1.890	0.887	4.539	0.033	6.617	1.163	37.639
LND	0%/0%–29%≥30% (0/1/2)	1.950	0.416	21.993	0.000	7.030	3.112	15.882
Mediastinal lymph nodes	Negative/positive (1/2)	−4.248	0.634	44.872	0.000	0.014	0.004	0.050
Constant	–	−5.502	1.709	10.369	0.001	0.004	–	–

### Construction of predictive mathematical model of abdominal lymph node metastasis

The variables with *P* < 0.05 were entered into the equation, and the exclusion criterion was 0.10. The regression coefficients were obtained by the maximum likelihood method, and the standard regression coefficients were calculated. The regression coefficients of these variables were used to establish a predictive mathematical model. The occurrence probability of abdominal lymph node metastasis was Y = e^x^/(1 + e^x^), in which X = −5.502 + 1.569 × lesion location + 4.269 × pN stage + 1.890 × vascular invasion + 1.950 × LND-4.248 × mediastinal lymph node metastasis. The variable assignments were shown in Table 2.

### ROC curve analysis of the efficacy of this predictive model in predicting abdominal lymph node metastasis

**Figure 1:**
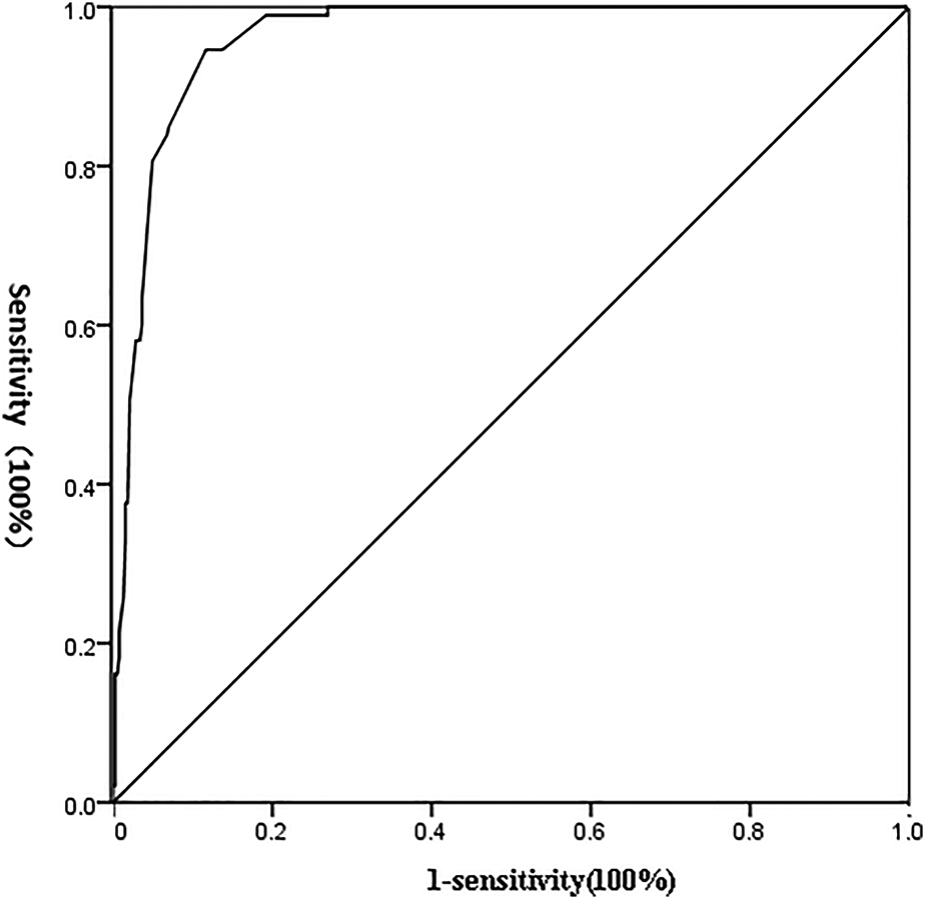
ROC curve of detection indicators independently affecting the occurrence of abdominal lymph node metastasis.

The predictive value of the occurrence probability of abdominal lymph node metastasis served as the detection variable, and the grouping was the state variable. The value of the state variable was set as 1, and the ROC curve was established. As shown in Figure 1, the area under ROC curve (AUC) of this model in predicting abdominal lymph node metastasis was 0.962 (95% CI, 0.946–0.977). The results suggested that this mathematical model had a significantly high value in predicting the occurrence of abdominal lymph node metastasis (*P* = 0.000); the sensitivity and specificity of prediction were 94.6% and 88.3% respectively.

### Analysis results of the patient's prognosis and the postoperative locoregional failure mode

**Figure 2:**
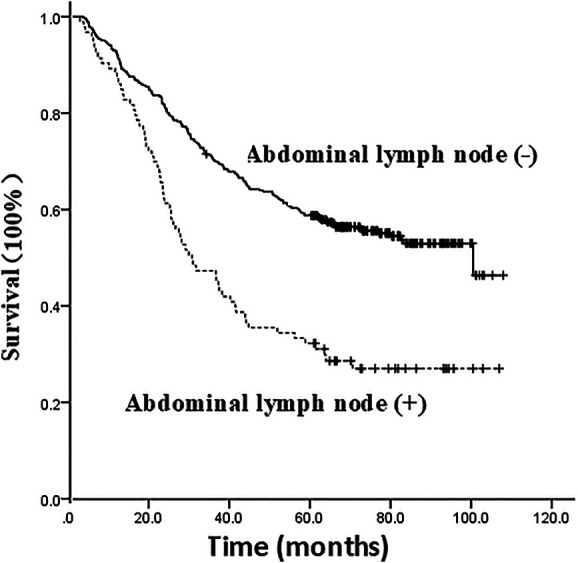
Survival curve of the impact of presence or absence of abdominal lymph node metastasis on the prognosis of patients.

The 1, 3, and 5-year survival rates of all patients were 91.6%, 66.0%, and 53.6%, respectively, with a median of 77.2 months (95% CI, 59.494–94.906). Pathological examination showed that the 1, 3, and 5-year survival rates of patients with positive and negative abdominal lymph nodes were 88.2%, 47.3%, 32.3%, and 92.5%, 70.5%, 58.8%, respectively, with a median of 72.2 months and 48.7 months respectively, suggesting a significant difference (X^2 ^= 29.178, *P* = 0.000), as shown in Figure 2.

In this group, postoperative pathology revealed that 374 patients had negative abdominal lymph nodes, including 113 (30.2%) with LRR, and 261 (69.8%) without LRR; postoperative pathology showed positive abdominal lymph nodes in 105 patients, including 50 (47.6%) with LRR, and 55 (52.4%) without LRR. Chi-square test indicated a significant difference in LRR rate between the two groups (X^2 ^= 11.063, *P* = 0,001).

In this group, supraclavicular lymph node and mediastinal lymph node are the common sites for postoperative LRR, as shown in Table 3. Postoperative pathology showed that during the follow-up of patients with positive (*n* = 105) and negative (*n* = 374) abdominal lymph nodes, abdominal lymph node recurrence was found in 8 cases (7.7%) and 5 cases (1.4%) respectively, suggesting significant difference (X^2 ^= 12.254, *P* = 0.000).

**Table 3:** Analysis results of local recurrent region of patients.

Recurrent region	Abdominal lymph nodes by postoperative pathology *N* (%)	
	Positive	Negative
Supraclavicular lymph node	9 (8.6%)	15 (4.0%)
Mediastinal lymph node	25 (23.8%)	73 (19.5%)
Anastomosis	3 (2.9%)	8 (2.1%)
Abdominal lymph nodes	7 (6.7%)	4 (1.1%)
Supraclavicular and mediastinal lymph node	4 (3.8%)	7 (1.9%)
Mediastinal and abdominal lymph nodes	1 (1.0%)	1 (0.3%)
Anastomosis and supraclavicular lymph node	0 (0.0%)	2 (0.5%)
Anastomosis and mediastinal lymph node	1 (1.0%)	2 (0.5%)
Anastomosis, supraclavicular and mediastinal lymph node	0 (0.0%)	1 (0.3%)

## Discussion

Abdominal lymph node area is one of the most common site for lymph node metastasis for patients with thoracic EC. Previous studies have shown that the occurrence probability of metastasis is 4.0%–57.0%. Currently, there has been no consensus regarding the effect of abdominal lymph node metastasis on the prognosis of patients (5–8). A retrospective analysis of abdominal lymph node metastasis in 913 patients with middle thoracic EC after surgery by Shen et al. (8) showed that the rate of abdominal lymph node metastasis in all patients was 4.1%, and abdominal lymph node metastasis was an important indicator of patient prognosis (*P* = 0.000). Chen et al. (9) analyzed 368 patients with middle thoracic EC after surgery, and the results showed that 58 patients (15.8%) had abdominal lymph node metastasis, and the 5-year survival rate of patients with abdominal lymph node metastasis and those with intrathoracic lymph node metastasis were 10.3% and 18.3% respectively (*P* = 0.029). Schomas et al. (10) analyzed 310 patients undergoing surgery for EC, and the results indicated that there was no significant difference in the prognosis of patients with respect to abdominal lymph node metastasis and mediastinal lymph node metastasis. In this study, postoperative pathology showed that the rate of abdominal lymph node metastasis rate in 479 patients with thoracic EC in a single center was 22.0%. In addition, the prognosis was significantly worse in patients with positive abdominal lymph nodes than in those with negative lymph nodes, with a median overall survival of 48.7 months and 72.2 months respectively (X^2 ^= 29.178, *P* = 0.000), which is similar to the results of the previous studies.

As is known to all, the stage N of EC is closely related to the prognosis of patients. Given the relationship between EC lesion location and the location of lymph node metastasis, the N staging system that integrates the location of lymph node metastasis and the number of metastases may have a greater significance in predicting the prognosis of patients (11). However, the TNM staging system for EC based on postoperative pathology focuses on the number of lymph node metastases in the N staging system, while the tumor location is not mentioned (12). The JESD-TNM staging system focuses on the impact of tumor location on lymph node metastasis, but neglect the effect of the number of lymph nodes on the prognosis of patients (13). The results of this study showed that the status of abdominal lymph nodes revealed by postoperative pathology was closely related to the total lymph node metastasis of patients after pathology. These related indicators included pN staging, LND and mediastinal lymph node metastasis. In order to explore the influencing factors of abdominal lymph node metastasis in TESCC, Li QM et al. (14) constructed a predictive model of abdominal lymph node metastasis of TESCC based on the risk factors, and analyzed the medical records of 443 patients undergoing surgery for EC. The predictive model indicated that for the regrouped patients who had no mediastinal lymph node metastasis or vascular invasion, the probability of abdominal lymph node metastasis at any tumor location was the lowest (11%); on the contrary, when there was vascular invasion and the total number of thoracic lymph node metastasis was ≥3, the probability of abdominal lymph node metastasis at any tumor location could be as high as 80%. Based on the findings of this study and previous studies, we suggest that for patients with positive mediastinal lymph nodes, clinicians should be alert to the possibility of abdominal lymph node metastasis before treatment.

Our study showed that vascular invasion was a risk factor for abdominal lymph node metastasis. Vascular invasion mainly includes lymphatic invasion, which is the beginning of lymph mode metastasis, and vascular invasion, which is closely related to the blood-borne metastasis of cancer cells. Previous studies have shown that vascular invasion is a risk factor for lymph node metastasis, tumor recurrence and poor prognosis in a variety of solid tumors (15–17). Currently, few studies are available on the relationship between vascular invasion and abdominal lymph node metastasis after EC surgery. The reason may be that after vascular invasion, cancer cells may penetrate the basement membrane of the relevant vessel wall and pass through its abundant cavity network to spread to the abdominal lymph nodes.

The results of this study indicated that tumor location was a risk factor for abdominal lymph node metastasis. The incidence of abdominal lymph node metastasis in patients with upper, middle and lower ESCC was 0%, 19.9% and 36.9%, respectively, suggesting significant differences. It was obvious that the shorter distance between the tumor and the abdomen was associated with the shorter distance of the tumor cells to invade the longitudinal lymphatic vessels in the esophageal wall, and the more likelihood of metastasis. Previous related studies have also shown that the location of lymph node metastasis in EC is closely related to the lesion location (18, 19). Upper thoracic EC is mainly related to metastasis to the cervical and upper mediastinal lymph nodes, lower thoracic EC is mainly related to metastasis to the lower mediastinal and abdominal lymph nodes, and middle thoracic EC is related to metastasis to the neck, chest mediastinum, and abdominal lymph nodes. Previous research results (9, 20, 21) indicated that stage T was an independent influencing factor for abdominal lymph node metastasis of TESCC. In theory, the deeper the tumor invasion, the greater the probability of lymph node metastasis. The results of this study suggested that pT stage was not significantly associated with abdominal lymph node metastasis in patients, which were similar to the findings by Li et al. (14). In their study, the probability of abdominal lymph node metastasis in patients with stages T2 and T3 was three times of those with stage T1. The negative result of our study may be related to the small number of patients with stage T1 (only 13 cases). Therefore, attention should be focused on to the possibility of abdominal lymph node metastasis in patients with late stage T.

Currently, surgical resection of EC is still the mainstay of treatment for early and locally advanced thoracic EC. Although advances in surgery, anesthetic techniques as well as improvements in perioperative management have reduced postoperative mortality, this reduction still fails to translated into the benefit of long-term survival of patients, which is primarily related to the high recurrence rate (34%–79%) and short remission period (median, 14 months) in patients with EC (22, 23). Therefore, postoperative adjuvant treatment, especially postoperative radiotherapy, is of great significance. Currently, there has been no consensus on the targets for postoperative radiotherapy, especially the range of clinical target volume (CTV). Based on the drainage of the longitudinal submucosal lymph nodes of EC and nodal skip metastasis, the range of CTV for postoperative radiotherapy in the past (24–26) consisted of bilateral supraclavicular area, mediastinal lymph node area and left gastric lymph node drainage area. Such a wide range of irradiation may effectively reduce the chance of local recurrence within the irradiation range (24), which, however, often causes severe gastrointestinal and systemic reactions simultaneously, thereby causing unnecessary radiation damage (27). Therefore, the optimal range of postoperative CTV should involve high postoperative recurrence areas, and reduce the incidence of unnecessary toxic and side effects, with respect to the following aspects (28–30): the range of lymph node dissection during surgery, postoperative location of recurrence, presence or absence of preoperative neoadjuvant radiotherapy and location of primary esophageal lesions. The results of this study showed that patients with positive abdominal lymph nodes undergoing surgery for thoracic EC are more likely to experience recurrence of abdominal lymph node than those with negative abdominal lymph nodes. Therefore, it is recommended that the abdominal lymphatic drainage region in these patients should be one of the targets for postoperative radiotherapy.

## Conclusion

In conclusion, patients with TESCC have a higher incidence of abdominal lymph node metastasis, and abdominal lymph node metastasis is a predictor of poor prognosis. According to the patient's clinicopathological risk factors, a predictive model of abdominal lymph node metastasis can be established, which can accurately predict its occurrence probability, and provide guidance for the choice of treatment schemes by clinicians. TESCC has a higher LRR rate after surgery, and several patients are recommended to undergo follow-up postoperative adjuvant therapy to reduce the recurrence. In addition, for patients with positive lymph nodes, it is recommended that the abdominal lymph nodes be included as one of the targets for postoperative radiotherapy.

## Data Availability

The raw data supporting the conclusions of this article will be made available by the authors, without undue reservation.
